# Gun violence, disability and recovery

**DOI:** 10.4102/ajod.v4i1.169

**Published:** 2015-04-20

**Authors:** Andrés Villaveces

**Affiliations:** 1 Senior Violence Prevention Specialist, World Bank Group, United States of America; 2 Injury Prevention Research Center, University of North Carolina, United States of America

The public health approach to the prevention of violence has been applied for almost the last 40 years. During this period, several studies have shown that in many countries where firearms are abundant and violence rates are high, the proportion of people dying because of firearms is high. Several studies have shown that the risk of suicide in the home due to firearms is increased when access to these devices is easy. The same occurs for homicide and for accidental deaths in the home. Whilst these studies show to a great extent the devastating effects of fatal gun injuries they do not sufficiently capture the human consequences of injuries occurring due to exposure to these devices.

*Gun violence*, *disability and recovery* is a timely publication that not only captures the devastating consequences of gun violence but also gives this problem a human touch by telling a wide variety of stories from different people in several countries, who share with the reader their lives after being injured by a firearm. In addition to providing narratives of survivors of gun violence or secondary survivors (families, friends of victims), the book highlights different perspectives that include professionals from the justice, health, and social sectors. Each chapter at the end contains spotlight sections that highlight specific cases from different countries.

Whilst these views are important to capture perspectives and indeed the direct human consequences of violence, this book goes well beyond sharing these narratives, and addresses the multiple needs associated to the victims: that include proper legal frameworks, rehabilitation resources and services – both physical and psychological – international standards, community and peer support, perspectives on human rights and victims’ rights, best practices and also a focus on perpetrators.

The first section, comprising chapters 1–5, focuses more on a variety of issues related to gun violence and its consequences. For example, chapter 2 of the book addresses a key factor which highlights victims’ rights and how these concepts have evolved from the social movements of the 1970s to the more complex compensation schemes that include tax levies, increased services and funding for victims and, in the case of armed conflict, the creation of the International Criminal Court (ICC). Using examples from different countries, a diversity of truth and reconciliation commissions and reparation schemes are highlighted that include restitution, compensation, rehabilitation, satisfaction, and guarantees of non-repetition. Finally the chapter addresses other strategies related to armed conflict such as vetting, amnesties and peace agreements.

Chapter 3 highlights physical injuries and trauma caused by gun violence and addresses not only the physical consequences to the body but also aspects of ballistics and how guns affect multiple organ systems. The chapter relates many consequences to the existence of proper trauma care systems and how these are key to increasing survival of individuals as well as reducing permanent disabilities. In chapter 4, the authors address the process of rehabilitation and what it entails, and highlight the importance of every step from arrival to health services to acute care measures, the existence of comorbidities and impairments due to gun injuries and the transition to life in the community. The focus is not only on the physical but also the psychological trauma and its consequences, and is essential and well linked to the need of proper mental health services. In the final sections of the chapter the authors focus on rehabilitation in the community, caregiving and the large amount of obstacles and costs associated with gun injuries. In every chapter the authors combine in a careful way the personal experiences learned through spotlight cases, with data about the problem and recommended practices and the links of these services to the need for proper legal and normative frameworks.

Chapter 5, the last one of this section, focuses on social protection. This chapter is key because it links single acts of violence to the devastating consequences for individuals, families and, in the end, for societies as a whole. The chapter highlights several typologies of social protection programmes. It addresses perhaps the most important factor, which relates to the huge economic consequences for victims of gun violence and their families. By linking the costs of rehabilitation to life after being victimised by a gun injury, this chapter highlights the risks of increased poverty, inequality and occupational impairments for individuals who have been affected by gun injuries. Importantly this chapter highlights efforts to address disabilities in the workplace but also the current existing gaps aimed at protecting victims in several countries. Importantly this chapter summarises very clearly six different social protection approaches for victims of gun violence and highlights their services, what and who they cover. A final call in this chapter is directed at ways to improve social protection programmes in different countries.

The second section of the book, from chapters 6–10, highlights all the previous issues addressed in section one, but contextualised in specific countries. From Guatemala to Somalia, to South Africa, to Canada, and finally India, these chapters address, in the following order, the magnitude of gun violence, the experiences of victims of violence and their rehabilitation procedures, and follow-up with contextualised explanations of contacts with the justice, health, and social protection systems of each nation. This section is especially important because it focuses on five countries that are very different and as such illustrates with real examples the challenges that are carefully laid out in the first section of the book.

The final chapter of conclusions and recommendations provides the reader with 13 key points aimed at a wide variety of disciplines and stakeholders and suggests possible strategies for improving prevention, care, rehabilitations, and protection for victims of gun violence. The recommendations highlight the need for improvement in trauma care and emergency services, rehabilitation and psychological support, caregiving and peer support, social protection, harmonisation of services including physical, psychological and social services, judicial responses to victims and to perpetrators. The chapter finally addresses the need for increased research, funding for services and strengthening of legal and normative frameworks for protecting victims’ rights including the passage of laws linking access to firearms to victims’ rights.

The authors of *Gun violence, disability and recovery* note that all of these approaches are relevant and this publication does an excellent job in linking personal experiences with the overall individual, family, community, and social responses that are needed to reflect the rights of victims of gun violence. It further improves these linkages with a rich review of the problem in different contexts that is further enhances by a wide variety of spotlight sections that complement all chapters. The book is a necessary resource for all those working to prevent and control gun violence as well as for those interested in victims’ rights.

